# Transcriptome-Wide m^6^A Methylome Profiling in Sorghum following GA_3_ Treatment under Salt Stress

**DOI:** 10.3390/ijms231810674

**Published:** 2022-09-14

**Authors:** Yanqing Wu, Jiao Liu, Guisheng Zhou

**Affiliations:** 1 Joint International Research Laboratory of Agriculture and Agri-Product Safety, The Ministry of Education of China, Institutes of Agricultural Science and Technology Development, Yangzhou University, Yangzhou 225009, China; 2 Jiangsu Provincial Key Laboratory of Crop Genetics and Physiology, Yangzhou University, Yangzhou 225009, China

**Keywords:** sorghum, gibberellic acid (GA_3_), salt stress, m^6^A methylome

## Abstract

Sorghum (“Jitian 3”) is a salt-tolerant seed cultivar used regularly in marginal lands, such as those with saline soils. Herein, we examined the potential of employing gibberellic acid (GA_3_) as an inducer of sorghum development during salt stress. Thus far, there have been no reports on the signaling network involved in the GA_3_-mediated regulation of sorghum development. In this study, we demonstrated that the stimulating properties of 50 mg/L GA_3_ on sorghum development was far superior to other GA_3_ concentrations under a 150 mM NaCl salinity condition. Furthermore, using methylated RNA immunoprecipitation sequencing (MeRIP-seq), we established an m^6^A methylation (m^6^A-M) profile in sorghum following exposure to 50 mg/L GA_3_. Overall, 23,363 m^6^A peaks and 16,200 m^6^A genes were screened among the GA_3_-treated and control leaves. These identified peaks were shown to be primarily enriched in the coding, as were the 3′- and 5′-untranslated regions. In addition, we employed m^6^A and transcript expression cross-analysis to identify 70 genes with differential transcript expression and simultaneous m^6^A-M. Intriguingly, the principal gene, *LOC8066282*, which is associated with *LOC8084853*, was shown to be intricately linked to the phosphatidylinositol signaling, which in turn regulates sorghum development and response to salt stress. This investigation presents a novel RNA m^6^A-M profile in sorghum, which may facilitate new insights into the underlying signaling behind salt stress resistance. This work will also benefit future investigations on foreign GA_3_ administration of sorghum.

## 1. Introduction

Salinity is a major disruptor of plant development, and it has contributed to a major decline in global crop production [1,2,3]. Elevated salt ion concentrations impair photosynthesis, cellular metabolism, and nutritional balance [4]. Moreover, emerging evidence has suggested that salt stress induces modifications in the lipid bilayer and membrane permeability [5,6,7]. Exogenous plant growth phytohormones administration is considered an efficient approach to alleviating negative salinity outcomes. Among the known phytohormones, gibberellin is a known inducer of plant development under salinity conditions [8,9]. Prior investigations revealed that exogenous gibberellic acid (GA_3_) usage accelerates seed germination, enhances seed salt tolerance, and alleviates the salt-mediated suppression of seedling development [10]. It has also been concluded that GA_3_ usage diminishes harmful salinity outcomes while enhancing salinity resistance in the mustard plant [11]. Sorghum (“Jitian 3”) is a salt-tolerant seed cultivar that has frequently been used in China over a minimum of 40–50 centuries in arid, semiarid, and water-logged locations. Despite some reports on the outcomes of foreign GA_3_ application in plants under salt stress, there are limited investigations on sorghum. Hence, the underlying signaling behind the GA_3_-mediated regulation of plant development under salt stress remains poorly understood.

Post- or co-transcriptional RNA modifications are ubiquitous among most eukaryotic cells, and they regulate numerous aspects of RNA metabolism. m^6^A is the leading modification in numerous eukaryotic transcripts, namely in yeast [12], plants [13,14,15], insects [16], and mammals [17]. m^6^A modification (m^6^A-M) is established via dynamic associations between its methyltransferases (the “writers”) (METTL3/14, WTAP, RBM15, and so on), demethylases (the “erasers”) (FTO, ALKBH5), and interacting proteins (the “readers”) (YTHDF1/2/3, YTHDC1, IGF2BP, and so on) [18]. Recently, m^6^A RNA methylation has received attention from scientists all over the world. In plants, m6A modulates multiple aspects of mRNA metabolism, including alternative polyadenylation and secondary structure, translation, and decay. These processes are critically involved in various plant growth and stress responses [19]. However, plant viruses disrupt m^6^A-M via interaction with key m^6^A pathway proteins, which, in turn, prevents modification [20]. To date, there have been no extensive reports on the m^6^A-M profile in sorghum. This information is crucial for building a complete understanding of the mechanisms behind plant growth and stress response.

Herein, we elucidated the outcome of salinity stress on sorghum during seedling development. We also identified the optimal GA_3_ concentration for alleviating most salt stress. In this aim, we conducted a greenhouse evaluation to examine the outcome of various GA_3_ concentrations on plant height, stem diameter, leaf area, dry matter, and the activities of superoxide dismutase (SOD), peroxidase (POD), and catalase (CAT), etc. Moreover, we performed a high-throughput Illumina sequencing analysis to elucidate the m^6^A-M profile in sorghum exposed to GA_3_ treatment under salt stress. Our findings will enhance our understanding of the role of GA_3_ in regulating salt tolerance in sorghum.

## 2. Results

### 2.1. Phenotypic and Morphological Variations of Sorghum (“Jitian 3”) with GA_3_ Treatment under Salt Stress

**Figure 1:**
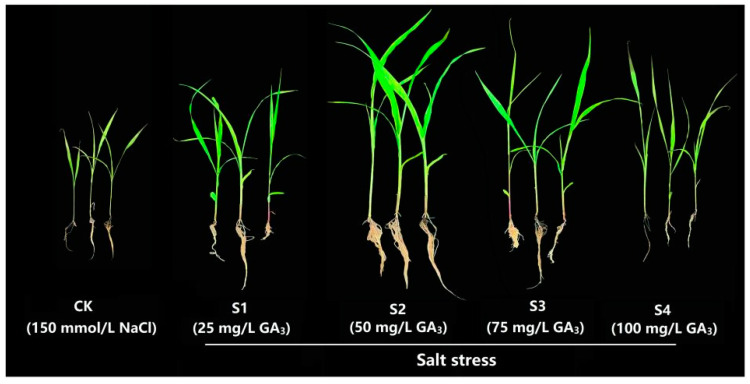
Phenotypic difference of the sorghum with exogenous gibberellic acid (GA_3_) exposure under salinity-stressed conditions. All sorghum plants were subjected to salt stress via the addition of 150 mM NaCl solution. CK represents the control group with salt stress. S1~S4 represent the experimental leaves with 25 mg/L GA_3_, 50 mg/L GA_3_, 75 mg/L GA_3_, and 100 mg/L GA_3_ treatment under salt stress, respectively.

**Figure 2:**
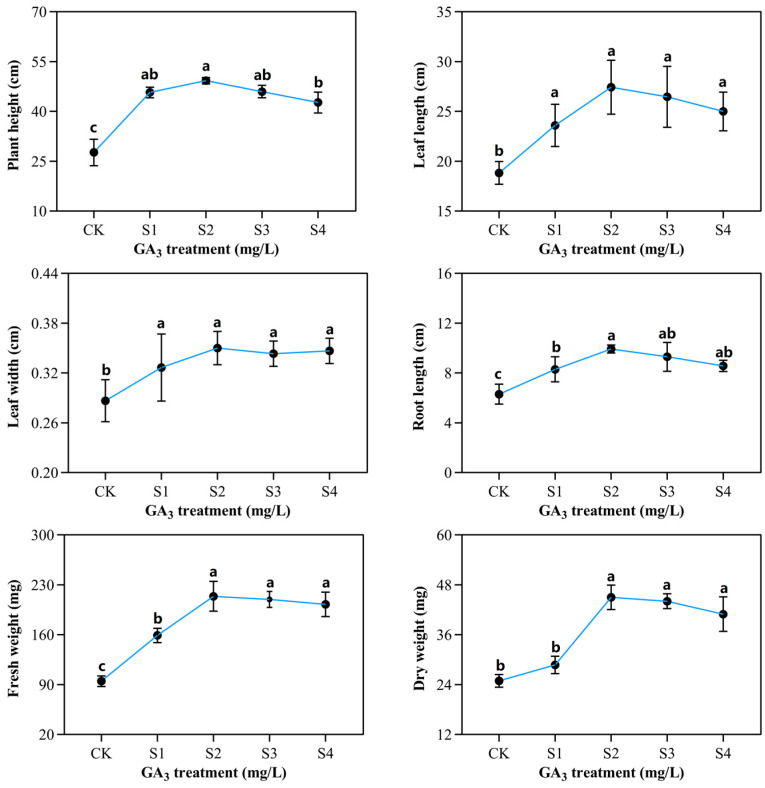
The outcome of varying exogenous GA_3_ concentrations on the phenotypic variations of sorghum seedling development under 150 mM NaCl exposure. CK—0 mg/L GA_3_; S1—25 mg/L GA_3_; S2—50 mg/L GA_3_; S3—75 mg/L GA_3_; S4—100 mg/L GA_3_. Six sorghum plants from GA_3_ treatment (n = 3) and the control group (n = 3) were used for phenotypic determination, including plant height, leaf width, fresh weight, leaf length, root length, and dry weight. All data are presented as the mean ± SD, Superscript different lowercase letters means significant difference (*p* < 0.05), Superscript same lowercase letters means significant difference showed no significant difference (*p* > 0.05).

To elucidate the outcome of exogenous GA_3_ on the phenotypic variations of sorghum (“Jitian 3”) under salt stress, we observed the phenotypic alteration of sorghum in the presence of varying GA_3_ concentrations. Sorghum growth was induced significantly on exposure to 50 mg/L GA_3_ (S2) (Figure 1), and further improvements in the values of other characteristics, such as plant height, leaf width, fresh weight, leaf length, root length, and dry weight were observed (Figure 2). GA_3_-treated plants exhibited remarkably higher height compared with controls. The largest plant height, 49.2 cm, was obtained with 50 mg/L GA_3_, and the lowest height, 27.6 cm, was obtained with 0 mg/L GA_3_. NaCl treatment significantly decreased both leaf length and width. The largest leaf length (27.4 cm) and width (0.35 cm) of the sorghum seedling were achieved with a GA_3_ concentration of 50 mg/L. The leaf length (8.8 cm) and width (0.28 cm) were the smallest when treated with 0 mg/L GA_3_. The root length was augmented at 25 mg/L GA_3_, and was diminished with 50 mg/L GA_3_ concentration. Sorghum seedlings treated with 150 mM NaCl and 50 mg/L GA_3_ demonstrated the largest root length (9.9 cm) compared with all other GA_3_ concentrations. With a rise in GA_3_ concentration, the fresh and dry weights of leaves, stems, and the entire plant were also gradually enhanced. We observed no discernible differences between the 50 and 100 mg/L GA_3_-treated outcomes, however, these results were markedly elevated compared with 0 mg/L GA_3_ treatment. The dry and fresh weights of sorghum seedlings’ leaves and whole plant rose initially, then diminished. The fresh and dry weights of the leaves, stems, roots, and the entire plant following 50 mg/L GA_3_ treatment was the highest, whereas seedlings treated with 0 mg/L GA_3_ showed the lowest fresh and dry weights. These data suggest that 50 mg/L GA_3_ had a positive effect on sorghum (“Jitian 3”) development compared to other GA_3_ concentrations under 150 mM NaCl salinity conditions.

### 2.2. Determination of Chlorophyll Contents and Enzymatic Activities in Sorghum (“Jitian 3”) with GA_3_ Treatment under Salt Stress

**Figure 3:**
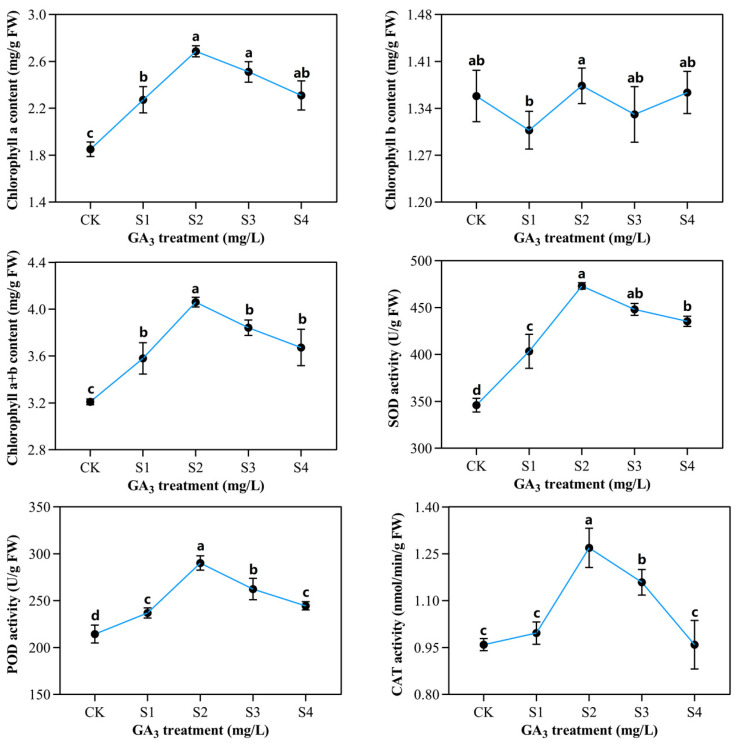
The influence of varying exogenous GA_3_ concentrations on chlorophyll content and antioxidant enzymatic activities of sorghum seedlings developed in the presence of 150 mM NaCl. CK—0 mg/L GA_3_; S1—25 mg/L GA_3_; S2—50 mg/L GA_3_; S3—75 mg/L GA_3_; S4—100 mg/L GA_3_. Six sorghum plants from GA_3_ treated (n = 3) and control leaves (n = 3) were used for the determination of chlorophyll contents, as well as superoxide dismutase (SOD), peroxidase (POD), and catalase (CAT) activities. All data are presented as the mean ± SD, Superscript different lowercase letters means significant difference (*p* <0.05), Superscript same lowercase letters means significant difference showed no significant difference (*p* >0.05).

Different GA_3_ concentrations affect chlorophyll content differentially under salt stress. As shown in Figure 3, chlorophyll content a was significantly affected by various NaCl and GA_3_ concentrations; however, there was no significant response to chlorophyll content b. In contrast, chlorophyll a+b content increased to 4.7 mg/g when treated with 50 mg/L GA_3_. In contrast, chlorophyll content decreased by 3.5 mg/g when treated with 100 mg/L GA_3_. We observed marked alteration in SOD, POD, CAT, SP, and proline levels after NaCl and GA_3_ treatments on all the sampling days. A measure of 50 mg/L GA_3_ markedly enhanced SOD, POD, and CAT activities compared to the controls. GA_3_ exposure also markedly enhanced POD activity relative to controls. With 150 mM NaCl and 50 mg/L GA_3_ co-treatment, POD activity rose to a maximum of 290 U/(g·min), and then decreased slightly (244 U/g·min). The CAT activity was markedly different among the five GA_3_ treatments. The maximum CAT activity was obtained after 50 mg/L GA_3_ exposure, and the lowest was obtained with 100 mg/L GA_3_. With increasing GA_3_ concentration, SOD activity was also augmented, reaching the highest level at 50 mg/L GA_3_, prior to a reduction at 75 mg/L and 100 mg/L. The maximal SOD activity (473 U/g FW) was achieved with 150 mM NaCl and 50 mg/L GA_3_ co-treatment, and the minimal activity (345 U/g FW) was obtained with 150 mM NaCl and 0 mg/L GA_3_.

### 2.3. Determination of Partial Organic Content in Sorghum (“Jitian 3”) with GA_3_ Treatment under Salt Stress

**Figure 4:**
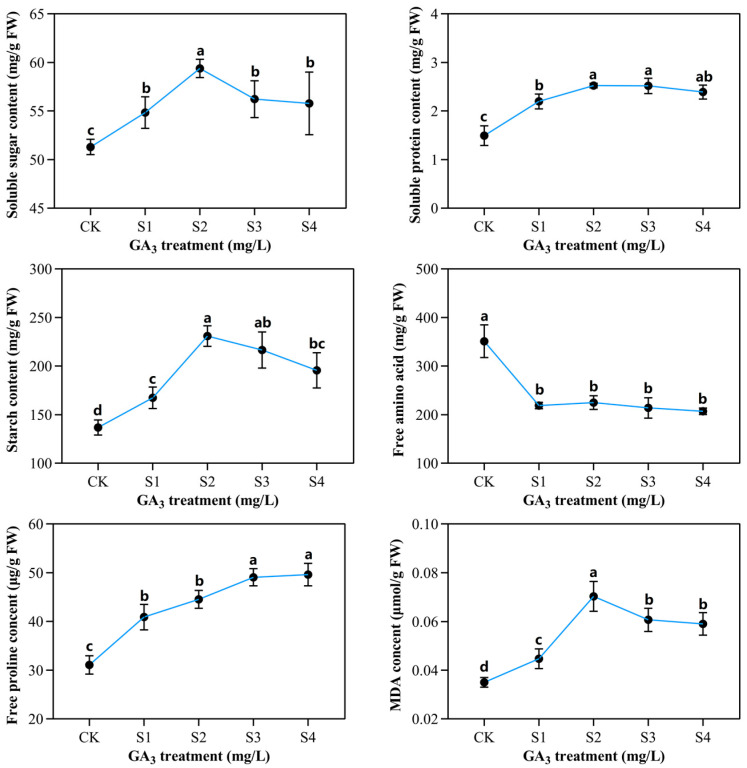
The outcome of varying exogenous GA_3_ concentrations on organic sorghum seedlings content developed under 150 mM NaCl conditions. CK—0 mg/L GA_3_; S1—25 mg/L GA_3_; S2—50 mg/L GA_3_; S3—75 mg/L GA_3_; S4—100 mg/L GA_3_. Six sorghum plants from GA_3_ treatment (n = 3) and control group (n = 3) were used for the determination of organic content, including soluble sugar, soluble protein, starch content, free proline, MDA levels, and free amino acid. All data are presented as the mean ± SD, Superscript different lowercase letters means significant difference (*p* < 0.05), Superscript same lowercase letters means significant difference showed no significant difference (*p* > 0.05).

To explore the outcome of exogenous GA_3_ on organic sorghum (“Jitian 3”) content under salt stress, we determined the levels of soluble sugar, soluble protein, free amino acid and starch content, MDA content, and free proline content (Figure 4). The soluble sugar and starch contents were markedly influenced by GA_3_ concentration and salinity. The GA_3_ and salinity stress promote soluble sugar aggregation. Relative to controls, the soluble sugar levels within leaves rose by 6%, 15%, 9%, and 8% at S1, S2, S3, and S4 concentrations, respectively. The starch levels within fresh Sorghum leaves were vastly different in the control versus the GA_3_-treated leaves. In fact, the starch concentration initially rose before diminishing with elevated GA_3_ concentrations. The maximum concentration (230.9 mg/L) was reached at S2, indicating that the GA_3_ environment strongly suppressed salt stress.

With a rise in salt content, the free proline and soluble protein concentrations rose considerably in sorghum seedlings on subsequent sampling days. The maximal soluble protein content (2.526 mg/g FW) was achieved at 50 mg/L GA_3_, and the minimal content occurred at 0 mg/L GA_3_. The free proline levels were considerably high (49.6 mg/g FW) following co-treatment with 100 mg/L GA_3_ and 150 mM NaCl.

At 150 mM NaCl, the highest free amino acid content was reported at 0 mg/L GA_3_. All GA_3_ concentrations failed to improve the sorghum seedlings’ free-amino-acid index when compared with untreated seeds. With increasing GA_3_ concentration, the MDA content was initially augmented, but then diminished, with 50 mg/L GA_3_ producing the largest MDA concentration under the reported NaCl concentration.

### 2.4. Effect of GA_3_ Treatment on Leaf Ultrastructure in Sorghum (“Jitian 3”) under Salt Stress

**Figure 5:**
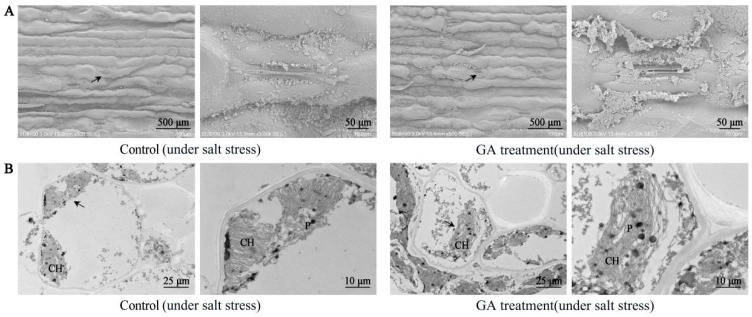
The outcome of GA_3_ treatment on the anatomical structure of sorghum leaves under salt stress. (**A**)—Influence of GA_3_ (50 mg/L) treatment on the epidermal structure and stomatal status of sorghum leaves under salt stress; (**B**)—influence of GA_3_ (50 mg/L) treatment on the cellular ultrastructure of sorghum leaves under salt stress; the figure on the right is an enlarged version of the area marked by a black arrow on the left figure. CH—chloroplast; P—plastoglobuli.

To further analyze the effect of 50 mg/L GA_3_ treatment on leaf ultrastructure in sorghum “Jitian 3”, we assessed mesophyll cells’ and sorghum leaf chloroplasts using TEM. As shown in Figure 5, under salt stress, almost all sorghum leaf stomata were closed in the control group; however, the stomatal aperture with the GA_3_ treatment was obviously wider than the controls (Figure 5A). TEM was adopted to assess the mesophyll cells’ ultrastructure in sorghum leaves (Figure 5B). The results revealed that, under salt and drought stress, mesophyll cells were severely deformed and irregularly shaped, chloroplasts gradually broke down, and part of the starch granular structure was disrupted. Compared with the control, the mesophyll cells’ ultrastructure of the GA_3_-treated leaves was more intact, as were the chloroplasts and starch granules.

### 2.5. m^6^A-M Profile within Sorghum under GA_3_ Treatment, as Evidenced by a Transcriptome-Wide m^6^A-seq

In this study, sorghum leaf tissues, treated with either 150 mM NaCl + 50 mg/L GA_3_ treatment (GL_A) or 150 mM NaCl treatment (GL_B), were used for m^6^A-seq assay, with three independent replicates per treatment. Using m^6^A-seq, we achieved about 48.48 Mb raw reads per sample, and approximately 89.45 million clean reads were mapped to the sorghum reference genome per sample (Supplementary Table S1). In terms of RNA-seq, about 55.90 Mb raw reads were obtained per leaf, and approximately 103.86 valid reads were mapped to the reference genome per leaf. The mapped reads proportion was between 88.97 and 94.87 (Supplementary Table S1). These results suggested that the high-quality sequencing data was suitable for use in subsequent analyses.

**Figure 6:**
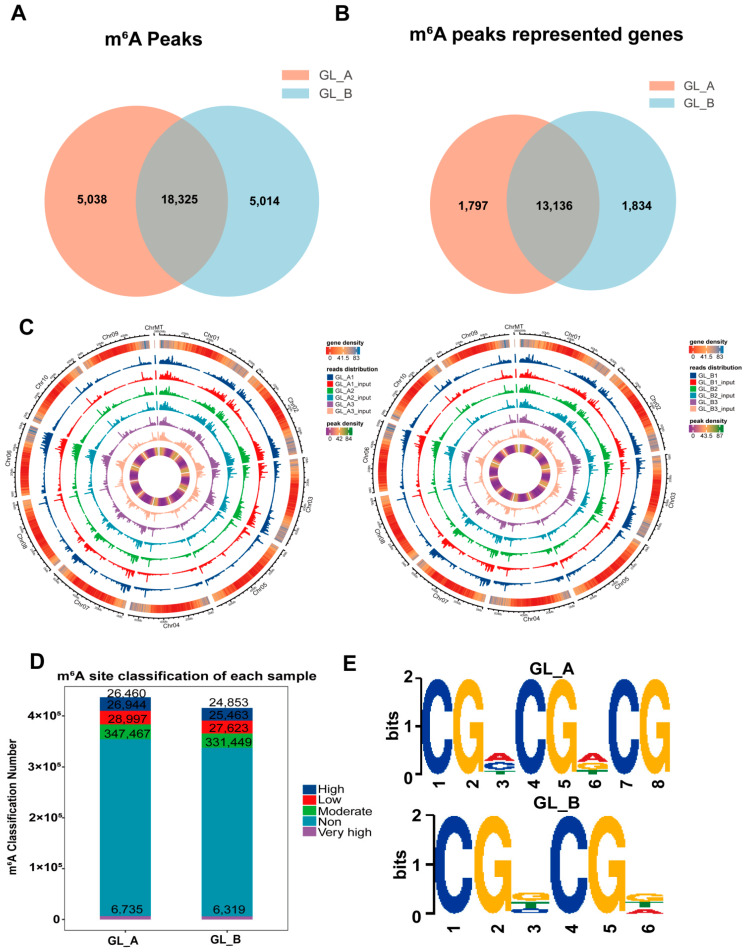
Transcriptome-wide m^6^A analysis in sorghum under GA_3_ treatment. (**A**) The quantity of common and distinct m^6^A peaks following GA_3_ (GL_A) versus control (GL_B) treatment. (**B**) A Venn diagram illustrating the m^6^A-associated mRNAs in both leaves. (**C**) m^6^A peak distribution across chromosomes in both leaves. (**D**) m^6^A site stratification in both leaves. (**E**) The top motif enrichments are related to m^6^A peaks in both leaves.

Employing the MeTDiff tool, we identified 23,363 and 16,200 m^6^A peaks in the GL_A and GL_B leaves, respectively (Figure 6A). Among them, 5038 and 5014 distinct peaks were noted in the GL_A and GL_B leaves, respectively, which reflected the difference between the examined leaves in the total m^6^A-M trends. Similarly, we annotated 14,933 and 14,970 genes in the GL_A and GL_B leaves, respectively (Figure 6B). Additionally, 18,325 peaks were persistently present in both leaves, and 13,136 genes (87.9% of all genes) were modified via m^6^A among the examined leaves. Furthermore, in terms of the genomic distribution, the m^6^A distribution analysis suggested that the m^6^A peaks were differentially distributed on the sorghum chromosomes in each group (Figure 6C). In addition, we also evaluated the differential m^6^A site stratification of each sample in both leaves (Figure 6D). The motif analysis revealed that both leaves harbored the classical m^6^A consensus sequences (Figure 6E). This finding added to the credibility of our m^6^A peaks and suggested the presence of a strong methylation modification system.

### 2.6. m^6^A-M Distribution in Sorghum under GA_3_ Treatment

**Figure 7:**
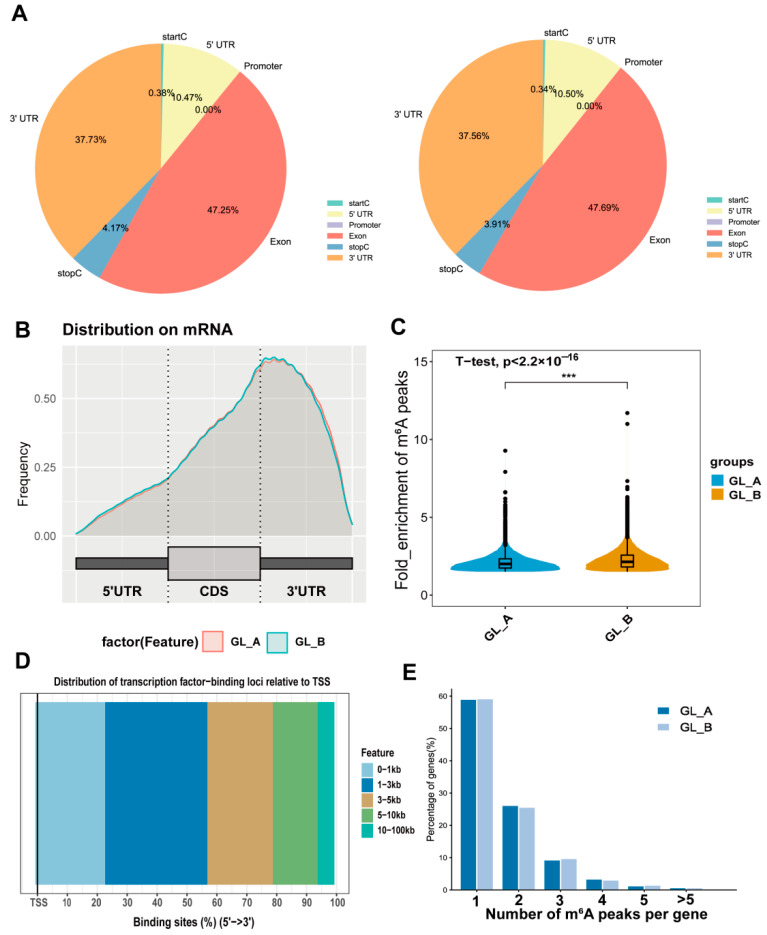
A summary of the m^6^A methylation (m^6^A-M) profile in sorghum under GA_3_ treatment. (**A**) Pie charts depicting the m^6^A peak distribution within transcripts. (**B**) Metagene plots illustrating the identified m^6^A peak regions within transcripts following GA_3_ (GL_A) and control (GL_B) treatments. (**C**) Violin plot revealing the m^6^A peak enrichment status distribution of individual leaves; *** *p* < 0.001. (**D**) The transcription-factor-binding loci distribution relative to TSS. (**E**) The quantity of m^6^A peaks per gene in the GL_A and GL_B leaves.

To explore the preferential m^6^A sites within transcripts, we assessed the m^6^A peaks profile in the sorghum transcriptome via coordination of the sorghum reference genome. The mRNA was separated into distinct regions as follows: the 5′-untranslated region (5′UTR), promoter, close to the start codon (startC), coding sequence (CDS) or exon, close to the stop codon (stopC), and the 3′UTRs. Based on our analysis, m^6^A-modified peaks enrichment was the maximum in the CDS (exon), followed by the 3′UTRs, 5′UTRs, and stopC region in the examined leaves (Figure 7A). The m^6^A peak density distribution exhibited similar trends in both the GA_3_-treated (GL_A) and control (GL_B) leaves (Figure 7B). However, the m6A peak enrichment degree varied significantly among the pairwise leaves (*p*-value < 2.2 × 10^−16^; Figure 7C). Furthermore, we analyzed the distribution of transcription-factor-binding loci relative to TSS, which was mainly located in the 1–3 kb region (Figure 7D). The m^6^A modified peak distribution of individual genes was assessed, and we revealed that most genes contained 1–3 m^6^A peaks in the GL_A and GL_B leaves (Figure 7E).

### 2.7. Differentially Methylated Gene (DMG) Evaluation

**Figure 8:**
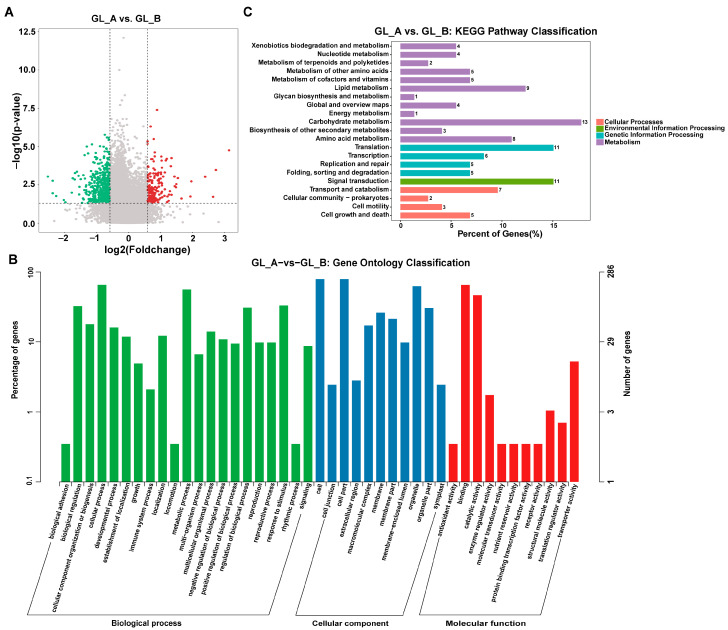
Differential m^6^A peaks of the pairwise comparison leaves. (**A**) Volcano plots depicting the differential peaks in the examined leaves. Red dots represent upregulated differentially methylated genes (DMGs) in GL_A group; green dots represent downregulated differentially methylated genes (DMGs) in GL_A group. (**B**) GO analysis for differentially methylated genes (DMGs). (**C**) KEGG network enrichment evaluation of DMGs.

To elucidate the role of m^6^A-M in sorghum following GA_3_ treatment, we conducted a pairwise comparison to identify DMGs. Relative to the GL_B group, 752 distinct DMGs were evident within 726 transcripts in the GL_A group (Figure 8A; Supplementary Table S2). Moreover, we carried out GO and KEGG analyses to establish the physiological roles of m^6^A-M in sorghum following GA_3_ treatment. Based on our GO analysis, all DMGs were primarily enriched in the organelle (ontology: cellular component (CC)), interacting, catalytic, and transporter activities (ontology: molecular function (MF)), as well as the developmental and metabolic process (ontology: biological process (BP)) (Figure 8B). KEGG evaluation revealed that the DMGs were markedly involved in the phosphatidylinositol signaling, lysine degradation, inositol phosphate metabolism, and so on (Figure 8C).

### 2.8. RNA-Seq-Based Screening of Differentially Expressed Genes (DEG)

**Figure 9:**
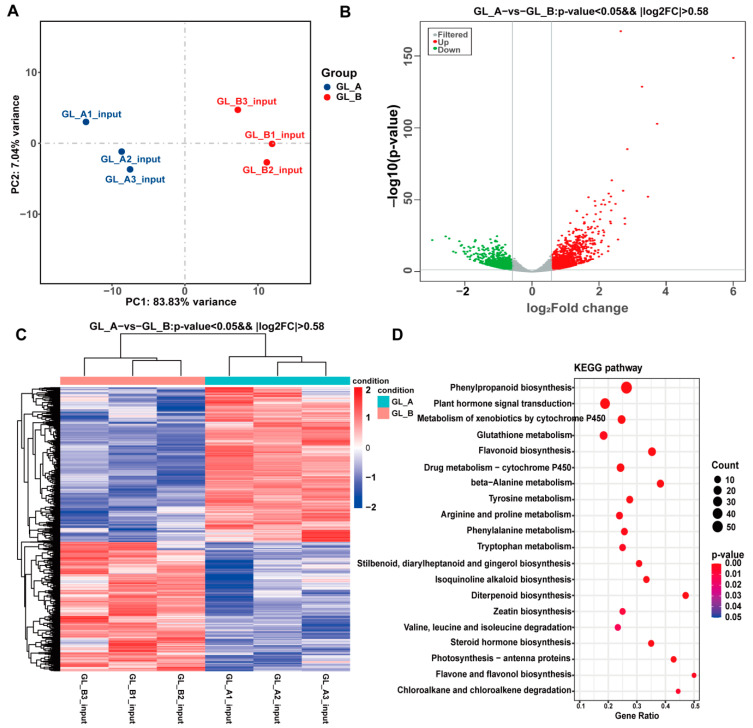
Analysis of differential expression, based on RNA-Seq data, between the analyzed leaves. (**A**) Principal component analysis (PCA) of Sorghum between the GA_3_-treated (GL_A, n = 3) and control (GL_B, n = 3) leaves. (**B**) Volcano plots depicting the differential gene expression between the examined leaves. (**C**) Heatmap plot of all DEGs. (**D**) KEGG network enrichment analyses of all DEGs.

In sorghum, we further analyzed the transcriptional differences between GA_3_ treatment (GL_A, n = 3) and controls (GL_B, n = 3) (Figure 9A). Using RNA-Seq, we identified a total of 2455 DEGs in the GL_A versus GL_B group (Figure 9B). Among them, 1335 were upregulated and 1120 were downregulated. The DEG hierarchical cluster profile is presented in Figure 9C. In addition, all DEGs were primarily enriched in 20 marked KEGG networks (*p* < 0.05, Figure 9D). Of note, most DEGs were strongly associated with the phenylpropanoid biosynthesis network and the plant hormone signaling network, followed by flavonoid biosynthesis and the tyrosine metabolic axis, and so on.

### 2.9. Conjoined Assessment of m^6^A- and RNA-seq Results

**Figure 10:**
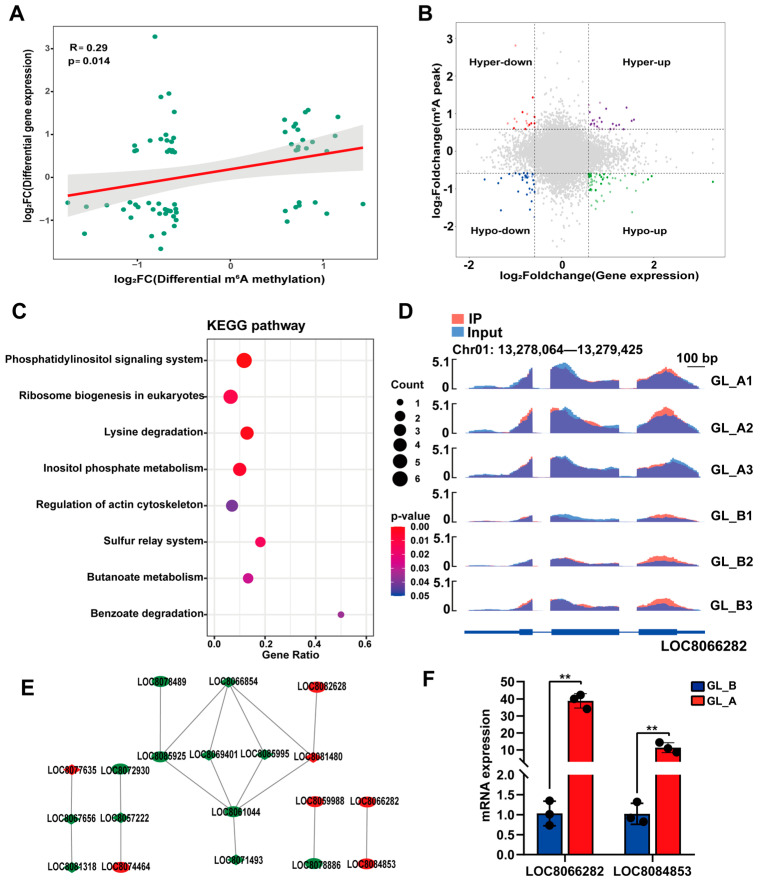
Combined analysis of the m^6^A- and RNA-seq data. (**A**) Log_2_FC (mRNA expression) versus log_2_FC (differential m^6^A methylation) dot plot depicting a strong and direct relationship between the sum of m^6^A-M and mRNA profile. (**B**) Four quadrant plots depicting DEGs with differentially methylated m^6^A peaks. Red dots represent the downregulated DEGs with hypermethylated m^6^A sites; Purple dots represent the upregulated DEGs with hypermethylated m^6^A sites; Green dots represent the downregulated DEGs with hypomethylated m^6^A sites; Blue dots represent the upregulated DEGs with hypomethylated m^6^A sites. (**C**) KEGG network enrichment analysis of genes depicting a considerable alteration in m^6^A and transcript expression. Different shades of colour represent the degree of significance. (**D**) m^6^A-modified LOC8066282 transcript data visualization. (**E**) Protein–protein interactions (PPIs) of genes with considerable alterations in m^6^A and mRNA transcript levels. Red annotated genes were upregulated DEGs in GL_A group, green annotated genes were downregulated DEGs in GL_A group. (**F**) Relative transcript expression, as evidenced by qPCR of critical genes in the examined leaves; ** *p* < 0.01.

To elucidate a possible association between m^6^A-M and gene expression, we conducted a cross-analysis of our m^6^A- and RNA-seq results. Figure 10A depicts a strong and direct association between DEMs and gene expression (p = 0.014, Spearman r = 0.29). Given that the absolute X and Y values were >2, the four-quadrant diagram analysis demonstrated a strong correlation between 682 DMGs and 660 associated genes (Figure 10B). Ultimately, 70 DMGs were strongly associated with transcript expression (*p* < 0.05, Supplementary Table S3). In addition, we employed all the identified genes in the KEGG network analyses (Figure 10C). Based on our analysis, most genes were markedly enriched in eight KEGG networks, namely the phosphatidylinositol axis, eukaryotic ribosomal biogenesis, lysine degradation, inositol phosphate metabolism, and so on. In the phosphatidylinositol axis, the *LOC8066282* transcript revealed a substantial hypermethylated peak (Figure 10D). Based on our STRING analysis, 19 genes were grouped into PPIs networks (Figure 10E). Among them, two DMGs (*LOC8066282*, *LOC8084853*) were chosen for analysis via RT-qPCR. We revealed that the selected DMG expression showed a tendency comparable to that of RNA-Seq (Figure 10F). Our findings suggested the critical roles of these candidate genes in the development of sorghum under GA_3_ treatment.

## 3. Discussion

Salinity is one of the most harmful abiotic stresses that inhibit plant growth and development. Similar findings have been described in which a low concentration of salt (<0.3%) had little effect on the growth of caster beans and even had some promotional effect, while a high concentration of salt (>0.6%) significantly inhibited the growth of seedlings [21]. Gibberellins (GA_3_ as the major type) are important plant hormones that are widely used to regulate plant growth during the whole life cycle of crop plants. A previous study showed that GA_3_ significantly enhanced plant growth and biomass yield of mustard (*Brassica juncea* L.) [11]. Thus, we identified the optimal GA_3_ concentration (50 mg/L) for alleviating salt stress by assessing its effect of on sorghum phenotypic and morphological variations, chlorophyll contents, antioxidant enzymes activities, organic content, and leaf ultrastructure. Plant height and leaf characteristics are important indicators of crop growth and development. Under salt stress, plant height, leaf area, and dry weight of sorghum plants are significantly reduced [22]. Gibberellins can regulate the osmotic potential of cells by accumulating intracellular substances that are harmless to protoplasts, thus resisting osmotic stress. Antioxidant enzymes play an important role in the scavenging of reactive oxygen species under salt stress. Under salt stress, reactive oxygen species (ROS) can damage functional macromolecular structures such as intracellular proteins and unsaturated fatty acids, leading to cell membrane lipid peroxidation [23]. After gibberellin application, the activity of SOD is increased, acting as the first line of defense for scavenging ROS. In this study, our findings showed that GA_3_ treatment with an appropriate concentration can enhance the protective enzyme activity in sorghum seedling leaves. Therefore, it is concluded in the present study that GA_3_ treatment increased the salt tolerance of sorghum seedlings, partly via improving the antioxidant system.

Some studies have shown that m^6^A modification (m^6^A-M) leads to mRNA instability at specific transcripts, which demonstrated the essential roles of m^6^A in various plant developmental processes and different tissues, including leaves and root tips [24,25]. MeRIP-seq is commonly employed to profile m^6^A-M in various species. Dominissini et al. successfully generated human and mice m^6^A-M patterns and explored transcriptome-wide m^6^A-M status in multiple species [26]. Other investigators characterized m^6^A-M patterns in *Arabidopsis thaliana* [27], *Oryza sativa* [13], and *Saccharomyces cerevisiae* [28]. Unfortunately, there have been limited studies on the sorghum m^6^A-M profile. Herein, we were the first to characterize the transcriptome-wide m^6^A-M landscape in sorghum leaves via MeRIP-seq. Our analysis demonstrated that the m^6^A-M sites were mostly enriched in the CDS and 3′UTRs, with somewhat reduced sites in the start/stopC regions. In mice, these peaks were more frequent across the internal region of modified transcripts, and they exhibited the following distribution frequencies: 37% CDS, 30% stopC, 20% 3′-UTR, and 13% TSS [29]. In *A. thaliana*, the m^6^A-M locations were mostly within the 3′-UTR and stop/startC regions [27]. Likewise, in *O. sativa*, most m^6^A-Ms were in the intergenic regions, with 70% prevalence in the CDS and 3′-UTR, and 20% in the intronic and 5′-UTR regions [13]. Based on these results, the m^6^A-M profile is species-specific. Generally, most mammalian and plant m^6^A-M locations are markedly enriched in the CDS and 3′-UTR regions, thereby indicating that these modification profiles are similar to the classical topological m^6^A patterns of mature transcripts. The m^6^A enrichment in these terminal regions is strongly correlated with RNA stability and signal transduction and may modulate targeting recruitments that regulate transcript transport or transfer. As the targets and mechanisms of m^6^A-mediated sorghum leaves development are poorly understood, mapping m^6^A targets in leaves under GA_3_ treatment will provide insights into m^6^A roles in salt stress.

Functionally, m^6^A-Ms control a myriad of biological processes, namely metabolism, differentiation, and disease etiology [30,31]. m^6^A-M profiles differ depending on tissues and environmental stimuli. Environment- and stimuli-based m^6^A-M profiles were previously examined in murine embryonic stem cells, embryonic fibroblasts, and preadipocytes [32,33]. Herein, we evaluated the m^6^A-M profile differences between GA_3_-exposed and control sorghum leaves. We demonstrated that genes that fell under DEGs and DMGs were closely related to metabolic networks such as the phosphatidylinositol axis and inositol phosphate. The leaf-specific m^6^A modified transcripts are mainly enriched in genes related to photosynthesis and respiratory metabolism, while the transcripts in roots are mainly enriched in genes that respond to stress, redox processes, and transporters. However, a large number of m^6^A modified transcripts in leaves are related to development, stress response, cell proliferation and differentiation [34]. Plants establish numerous cellular and molecular networks to adapt to salt stress. These include transport system activation, metabolic regulation, and transcriptional response modulation [35]. The phosphoinositol axis serves an essential function in plant development and their response to environmental stress [36]. In terms of the identified m^6^A-modified genes, our RNA-seq and qPCR co-analyses revealed that *LOC8066282* was a significant DEG in GA_3_-treated sorghum. Moreover, *LOC8066282* associates with *LOC8084853* in the generated PPI network. *LOC8066282* contributes to the phosphoinositol axis, thereby regulating salt stress. This indicates that both *LOC8066282* and *LOC8084853* likely serve essential roles following GA_3_ treatment. However, limited investigations assessed sorghum *LOC8066282.* Thus, it is critical to extensively examine the relationship between *LOC8066282* levels and sorghum development using Cas9-knockout experiments. In this study, we provided a comprehensive review of the advances in the roles of gibberellins on the growth, physiology, and molecular mechanisms of sorghum under salt stress, intending to better understand the roles of gibberellins in improving the yield and quality of sorghum grown in saline soils.

## 4. Material and Methods

### 4.1. Plant Materials, Growth Condition, and GA_3_ Treatment

The homogeneous and healthy sorghum seed cultivar was “Jitian 3”, a gift from the Agricultural Research Institute in Hebei Province. A factorial format was used to design the experiment, and it included one salinity concentration, 150 mM NaCl, and five GA_3_ concentrations, which were 0, 25, 50, 75, and 100 mg/L. Each treatment was replicated thrice. All sorghum seeds were immersed in varying GA_3_ concentrations for 12 h without light at 25 ℃. Subsequently, homogeneous and healthy sorghum bean seeds were selected and soaked with 0.1% NaClO solution for 15 min for disinfection, and then rinsed with purified water three times. Afterwards, the seeds were put in a Petri dish and soaked with 1/2 concentration of Hoagland solution for 48 h for pre-germination. Seeds with comparable bud lengths were planted in a plastic tray (50 cm long; 30 cm wide; 5 cm high) with air holes on the bottom. Next, all pots (5 cm in top diameter and 2.5 cm in bottom diameter) were packed with quartz sand. We planted a maximum of three seeds per pot at a depth of 3 cm. All data and sample collection were randomly performed at 40 days from one of three replicates per treatment. Lastly, the collected samples, along with 0 mg/L and 50 mg/L GA_3_ controls, were assessed and further evaluated via RNA sequencing.

### 4.2. Morpho-Physiological Assessments

We collected six sorghum plants from GA_3_ treatment (n = 3) and controls (n = 3) and carefully rinsed them with distilled water. Next, the true leaf length and width, plant height, and root strength measurements were recorded. The roots were cleaned with distilled water before separation into roots, stems, and leaves. The fresh weight was recorded. Then, the leaves were oven-dried at 70 ℃ for 72 h until a constant weight was achieved for biomass evaluation. At this point, the dry weight was recorded.

### 4.3. Chlorophyll Contents

To examine the chlorophyll composition, we followed the Arnon method, with slight modifications. In short, leaves were overnight (ON) immersed in 10 ml of 80% acetone without light, then 0.5 g of the leaf samples were isolated, followed by 10,000× *g*, 5 min centrifugation, and absorption (abs) measurement (supernatant) at 645 and 663 nm via a spectrophotometer. To determine chlorophyll levels in mg g^−1^ FW, we utilized the following formulas:

Total-Chl = [20.2(A645) + 8.02(A663)] × (V/1000W),

Chl-a = [12.7(A663) − 2.69(A645)] × (V/1000W), and

Chl-b = [22.9(A645) − 4.69(A663)] × (V/1000W).

where A645 and A663 represented chlorophyll abs at 645 and 663 nm, respectively; V represented the total extract volume; W represented the fresh leaf weight.

### 4.4. Antioxidant Enzymatic Activities Determination

To determine the enzymatic activity, 0.5 g of fresh sorghum seedling leaf was sliced into smaller portions, then combined with quartz sand and 5 mL 50 mM/L phosphate-buffered solution (pH = 7.8). This mixture was then crushed on ice prior to centrifugation at 15,000 r/min for 20 min at 4 °C. The resulting supernatant was maintained at low temperature until SOD, POD, and CAT activities were assessed via corresponding kits (Suzhou Keming Biotechnology Co., Ltd., Suzhou, China) following kit directions.

### 4.5. Evaluation of Soluble Sugar, Soluble Protein, Free Amino Acid, and Starch Content

The soluble sugar content was assessed via the anthrone colorimetry procedure. Soluble protein was evaluated via the coomassie bright-blue G-250 staining technique. Free amino acid was detected via the ninhydrin chromogenic method. Starch content was detected by the 3,5-dinitrosalicylic acid method.

### 4.6. Evaluation of MDA and Proline

The leaf MDA concentration was measured via the thiobarbituric acid reaction, as reported by Heath and Packer (1968) [37]. Proline quantification employed a procedure reported by Bates et al. (1973) [38], with slight modifications.

### 4.7. Anatomical Observation

The Zhao et al. (2020) study was referenced for our anatomical evaluation [39]. Fresh leaves were sliced into smaller portions with approximate dimensions of 1 × 1 cm, prior to fixation in 2.5% glutaraldehyde, and maintained at 4 °C for ≥4 h. Then, the leaves were rinsed thrice in 0.1 M phosphate buffer for 15 min each, prior to a secondary fixation in 1% osmium tetroxide for 4 h. Subsequently, they underwent dehydration in 100% acetone and acetone containing anhydrous sodium sulfate for 15 min each. Following embedding in Spurr resin, the sections were sliced, followed by double-staining with uranyl acetate and lead citrate. Lastly, mesophyll cell and chloroplast observation and imaging were performed under a transmission electron microscopy (TEM) (HT7700, HITACHI, Tokyo, Japan).

### 4.8. RNA Extraction, Library Generation, and Sequencing

Total sorghum leaf RNA was isolated from GA_3_-treated (GL_A, n = 3) and control leaves (GL_B, n = 3) using Trizol (Invitrogen, Carlsbad, CA, USA), and RNA quality was determined via a NanoDrop ND-1000 spectrophotometer (Thermo Fisher Scientific, Waltham, MA, USA). Subsequently, MeRIP- and RNA-seq were carried out by Oebiotech Corporation (Shanghai, China), employing protocols from prior publications [40]. In brief, the GenSeq™ m^6^A-MeRIP Kit (GenSeq Inc., Shanghai, China) was employed for m^6^A RNA immunoprecipitation (IP), following kit protocols. The IP and control samples were then utilized to generate an RNA-seq library using the NEBNext^®^ Ultra II Directional RNA Library Prep Kit (New England Biolabs, Inc., Washington, DC, USA). An Agilent 2100 Bioanalyzer (Agilent Technologies, Inc., Santa Clara, CA, USA) was employed for quality confirmation of the prepared libraries, and an Illumina HiSeq 4000 sequencer (Illumina, Inc., San Diego, CA, USA) was employed for library sequencing with 150 bp paired-end reads. Lastly, the raw high-throughput m^6^A and RNA-seq data were uploaded into the NCBI SRA database (BioProject ID: PRJNA858555).

### 4.9. m^6^A Sequencing Data Analysis

Following sequencing, the raw data (raw reads) in fastq format were initially processed with the Trimmomatic software [41]. Clean data (clean reads) were achieved via removal of reads with adapter, ploy-N, or reduced-quality data from raw information. In the meantime, the SortMeRNA software was employed for ribosomal RNA read elimination [42]. Next, the clean library reads underwent alignment against *Sorghum bicolor* reference genome (https://www.ncbi.nlm.nih.gov/genome/?term=sorghum, accessed on 15 February 2022) using HISAT2 [43]. To determine the methylated RNA IP sequencing data quality, we employed the Guitar R package [44] and deeptools software [45]. The m^6^A-enriched peaks in each m^6^A IP sample were recognized via the MeTDiff software with matched controls [46]. The peaks were stratified by intersection with gene architecture with the ChIPseeker [47]. Differential m^6^A-M rates between GA_3_-treated and control plants were assessed with diffReps [48]. Significant peaks were defined as log2|fold change| ≥ 1 and FDR ≤ 0.05.

Next, we evaluated the m^6^A-peak-distribution profile, based on various functional regions (TSS, CDS, stopC, 3′-UTR, 5′-UTR), and the m^6^A peaks throughout the entire mRNA were established with the Integrative Genomics Viewer tool (http://software.broadinstitute.org/software/igv/, accessed on 20 March 2022). Relevant motifs were screened via MEME suite (http://meme-suite.org/, accessed on 8 May 2022) and DREME (http://memesuite.org/tools/dreme, accessed on 8 May 2022). The HTSeq program (v0.9.1) was employed for raw mRNA-seq read data retrieval, which was then adjusted via edgeR. Lastly, we conducted the GO (www.geneontology.org, accessed on 16 May 2022) and KEGG network (www.genome.jp/kegg, accessed on 16 May 2022) enrichment analyses of differentially expressed mRNAs and differentially methylated genes (DMGs).

### 4.10. Quantitative Real-Time PCR (qRT-PCR) Analysis

qRT-PCR was utilized for mRNA assessment using BIO-RAD CFX Connect™ Optics Module (Bio-Rad, Hercules, CA, USA). Isolated leaf RNA (1 μg) was converted to cDNA in a 10 μL reaction using the superscript first-strand synthesis system (PrimeScript^®^ RT Reagent Kit With gDNA Eraser, TaKaRa, Osaka, Japan). *SORBI* served as the endogenous control. All targeted primers were synthesized by Beijing Qingke Biotechnology Co., Ltd. (Beijing, China) and are summarized in Supplementary Table S4. SYBR^®^ Premix Ex Taq™ was employed for qRT-PCR reactions (TaKaRa, Osaka, Japan). The PCR cycles were adjusted as described below: 55 °C for 2 min, 95 °C for 30 s (denaturation), 40 cycles at 95 °C for 5s, 55 °C for 15 s, and 72 °C for 30 s. Relative gene expression was computed using the 2^−ΔΔCt^ comparative threshold (Ct) formula.

### 4.11. Statistical Analysis

One-way ANOVAs in SPSS, version 25.0 (IBM, Armonk, New York, NY, USA), were employed for all data analyses. A *p*-value < 0.05 was deemed as significant.

## 5. Conclusions

Herein, we established an m^6^A RNA-methylation profile using high-throughput sequencing. Our work highlights the critical physiological roles of epigenetic alterations in sorghum. Using combined m^6^A-RIP- and RNA-seq analyses, we demonstrated the influence of 70 DMGs in modulating gene expression. Moreover, we identified the node genes (*LOC8066282*, *LOC8084853*), which have principal roles in the modulation of sorghum development. Additional investigations are warranted to validate the identified DMGs in sorghum.

## Data Availability

The raw high-throughput m^6^A and RNA-seq data were uploaded into the NCBI SRA database (BioProject ID: PRJNA858555).

## Supplementary Materials

The supporting information can be downloaded at: https://www.mdpi.com/article/10.3390/ijms231810674/s1.

## Abbreviations

GA_3_	gibberellic acid
m^6^A-M	m^6^A methylation
SOD	superoxide dismutase
POD	peroxidase
CAT	Catalase
UTR	untranslated region
stopC	stop codon
startC	start codon
CDS	coding sequence
DMGs	differentially methylated genes
DEGs	differentially expressed genes
PCA	principal component analysis
qRT-PCR	quantitative real-time polymerase chain reaction
